# Treatise on Intermittent, Remittent, and Continued Fevers of Hot Climates and Marshy Countries, Followed by Researches on the Therapeutic Employment of Arsenical Preparations

**Published:** 1842-07

**Authors:** 


### Art. III.

*Traité des Fièvres Intermittents, Remittents, et Continués, des Pays Chauds, et des Contrées Marécageuses, suivi de Recherches sur l'Emploi Thérapeutique des Préparations Arsénicales.* Par J. C. M. Boudin. —*Paris,* 1842. 8vo, pp. 336.

*Treatise on Intermittent, Remittent, and Continued Fevers of Hot Climates and Marshy Countries, followed by Researches on the Therapeutic Employment of Arsenical Preparations.* By J. C. M. Boudin. —*Paris,* 1842.

M. Boudin, in his capacity of a physician of rank in the French army, has, as he informs us, enjoyed many opportunities of observing the effects of marsh miasmata in several parts of France, Germany, Spain, Greece, and the Algerine districts of Africa. A summary statement of the fruits of his study in those countries is all that his volume professes to give, for M. Boudin condemns the custom into which his contemporaries have fallen of augmenting the size of their publications by loading them with numerous details. He makes a better apology for the omission, which will be deemed serious by many, when he hints at the long and painful peregrinations during which his notes were made, than when he professes to consider the narration of cases, and the record of particulars, unnecessary and inconvenient, because they afford no better security against dishonest sophistication, while they consume the time and patience of the reader. As this opinion is not confined to M. Boudin, we may remark, in passing, that detailed reports and observations are to be regarded less as tests of an author's probity, than of his means and methods of investigation, and of his capacity to observe and reason correctly, for it is to be hoped that intentional misrepresentation does not enter, in any considerable degree, into the sources of error in medicine.

M. Boudin's first chapter contains his definition of marsh poisoning (l'intoxication des marais), or limnhemic affections, (affections limnhémiques, from λιμνη, marsh, and αιμα, blood;) under which term he comprehends all maladies endemic in marshy countries, and described by authors under the names of intermittent, remittent, pseudo-continued, masked, and pernicious fevers, &c., a system of nomenclature which he conceives to have thrown a very great obstacle in the way of a correct understanding of the nature of the diseases of which he treats. Allowing the old designations to which he objects to be imperfect, we cannot see in them the amount of evil which he deplores, since the various disorders which they signify have been amply acknowledged as traceable to a common cause. Nor is it an easy matter to perceive how the general denomination which he proposes to substitute will "reduce the types and forms to their true value, destroy the factitious barrier erected by error between intermittent and continued fevers (of marshes), and conduct, by establishing a consanguinity between the different forms of the same disease, to a more rational treatment," (p. 31;) seeing that the true value of types and forms may be understood without a unity of name, and that consanguinity may be fully established and admitted, though the patronymics may be various.

His second, third, fourth, and fifth chapters contain, in the space of thirty-two pages, his views of the etiology of marsh diseases, the action of heat and of cold in producing them, the nature of miasm, and the modes of its introduction into the system. They contain no novelties of any value.

The sixth chapter treats of the antagonism of the palludal poison to certain pathological conditions, and more especially to the tubercular diathesis.

"The rarity and even complete absence of pulmonary consumption in certain localities is an incontestible fact, of which I shall not undertake the demonstration; but, if all physicians agree in recognizing it, it is still necessary that this remarkable phenomenon should be ascribed to its proper cause." (p. 72.)

With this sentence the author commences his demonstration of this proper cause residing in the marsh poison, and seems to speak of the opinion as hitherto unknown to the world, a conviction in which he is doubtless confirmed by the testimony of the "Société Royale de Médecine de Marseille." That learned body, in reference to this discovery, declares that "The observation which follows is one of great importance, and our colleague has all the merit of it; for no one that we know of has made it before him." (p. 330.) Perhaps some excuse for the ignorance of both parties on this point might be found in the circumstance that the essay of Dr. Wells, in which the doctrine was originally broached above thirty years ago, was published in a periodical work which did not continue long in existence;* were it not that Dr. Wells's doctrine has been noticed by many systematic writers on consumption. It is, nevertheless, true that the observations of that ingenious and able physician, though founded mainly on the statements of incompetent witnesses, on floating rumours, and general impressions, considering the importance of the subject, and the curious coincidence if not the respectability of the testimony which was adduced, merited more consideration and enquiry than have been bestowed upon them. The additional statements which are adduced by M. Boudin in support of the doctrine, though destitute of that precision and detail which alone can serve as the solid foundation of a general conclusion, are not altogether without interest.

"The action, sometimes prophylactic, sometimes palliative, of certain countries on the tubercular diathesis, has almost always been attributed to the influence of *méridionalité* of geographical latitude, or of temperature, if the

* Transactions of a Society for Improving Medical and Surgical Knowledge, vol. iii. 1812.

term be preferred. But it is requisite that observation should justify such an opinion, and to be convinced of this, it is sufficient to reflect that, if the febriferous Algeria excludes pulmonary consumption, the delta of the Rhine in Holland does so equally, whilst this malady, almost unknown in the marshy part of the Romagna, from the mouth of the Arno to Terracina, rages with the most cruel intensity at Naples, Malta, Gibraltar, and Corfu...........

"If tubercular consumption is rare in the marshy part of the north of Italy, to compensate for this, it exercises great ravages in certain southern countries of that peninsula. According to a statistical work, presented by M. Journé to the Royal Academy of Medicine of Paris, which he had framed at Naples from 1833 to 1837, 695 deaths occurred from pulmonary consumption, that is, 1 in 2·34 of the whole mortality. I have established many times the same contrast in the north of Africa. The rarity of diseases of the chest at Algiers is such, that it has happened to me to visit many hundreds of fever patients, without having had occasion to practise in a single instance auscultation or percussion of the respiratory organs. Among a total of 12,853 patients whom I have treated either in the army of Africa or at the Lazaretto of Marseilles (after their return from Africa), I have met with only 31 cases of consumption, of whom 25 had incontestably been affected with tubercle before having embarked for the Morea or Algeria.... ........

"We read in a letter addressed to the Academy of Medicine of Paris, 23d October, 1833, by M. Moreau, then physician to the army of Africa, the following passage, which constitutes a new proof in support of my opinion : 'I join to my letter a numerical table of the affections which were presented in the service with which I was charged at the military hospital of Bona; it comprehends a period of two years and a half. It thence appears that among 6,245 patients, 12 only figured as attacked with consumption, and that among 250 deaths, there were but 6 from that disease. .............. The affections of the thoracic organs are presented so rarely to my observation in Africa, that their study is become very necessary to me, and I am come to Paris in order to follow the instructions and the practice of those great masters who treat those diseases daily. ............. In short, my experience and my practice in Africa authorize me to conclude: 1st, that consumption is extremely rare among the inhabitants of that country; 2d, that Europeans are rarely affected by it; 3d, that the progress of the disease is retarded (enrayée) in consumptive Europeans transported to Africa.'" (pp. 72-5.)

M. Boudin adds, that while consumption is thus proved to be extremely rare in the essentially febriferous part of Algeria, the immunity from the tubercular diathesis appears to decrease in proportion as the country becomes more healthful in respect to the marsh diseases; so that a district often farther south, but less marshy than the coast, predisposes so much the more to consumption the less it is subject to fevers. "Thus, at Constantine," says M. Bonnafont (Géographie Médicale de l'Algérie), "where the fevers are both less serious and less frequent than at Algiers, a greater number of individuals affected with diseases of the chest, with scrofula, rachitis, &c., diseases almost unknown at Algiers, are remarked." (p. 77.)

Such is nearly the whole amount of M. Boudin's facts in support of his opinion of the beneficial agency of marsh exhalations in consumption. They are obviously extremely inconclusive and defective from the want of accurate statistical data and various details necessary to enable us to judge both of the value of the facts and of the other differences which may have existed between the localities which he contrasts, besides the existence and absence of marshes.

Before quitting this subject we shall adduce a few facts of an opposite kind to those on which the above opinion rests. On turning to the statistical reports of sickness, &c. in the British army, we find that, in our West India possessions, of the more serious kind of diseases which affect the European troops, the most prevalent are those which M. Boudin has placed in the relation of antagonism, the so-called marsh fevers, and diseases of the lungs, of the latter of which consumption forms nearly one half. Among the black troops, the supposed protective power of miasmata against this class of diseases is still less observable, more dying annually from thoracic affections, among which consumption forms a very large proportion, than among the same number of troops in the United Kingdom by all diseases together. Yet the black troops are more constantly exposed to miasmata than the Europeans are. In Canada, the proportion of marsh fevers in the Upper compared with the Lower province has been found as 178 to 26, or indeed much larger, since the greater number of cases reported as occurring in Lower Canada had originated in the Upper province; yet the returns, so far as they go, render it probable that "the influence of consumption would be equally manifested in both." It still remains therefore to be discovered on what the great rarity of thoracic diseases among the troops in Algiers depends.

M. Boudin traces cholera, plague, and yellow fever to marsh poison, notwithstanding the ample testimony which exists of the frequent occurrence of those maladies in circumstances which exclude the possibility of the operation of that agent. "These three forms of disease," he says, with singular ignorance, "show themselves constantly preceded, accompanied, and followed by intermittent fevers." (p. 161.) There is a kind of cholera, doubtless, very common in the marshy districts of all hot countries; and yellow fever often affects localities of the same kind, as does also plague; but their frequent occurrence in situations far removed from everything like marsh is so well known, that M. Boudin's assertion serves but to illustrate the power of preconception on men of moderate understanding. In respect to plague, it has been asserted by Van Swieten, that intermittents are so opposite to it in their nature, that while other diseases showed a tendency to pass into that distemper when it was prevalent, tertian never degenerated into it. The plague of London in 1665 occurred at a time, as attested by Sydenham, when the city was unusually free from agues. In Egypt, too, it is well known that plague habitually ceases as an epidemic about Midsummer, the very time at which intermittents begin to prevail.

The nature and seat of marsh maladies are discussed in the tenth chapter. The author chooses to select the blood as essentially the seat of those disorders. In that fluid the miasm may lurk for a long period, and even without ultimately affording any evidence of its presence; and it is to this preoccupation of the blood that he ascribes the supposed protection against the tubercular *virus.* A curious instance, if there be no mistake, of the contamination of the blood by marsh miasm is given at p. 193:

"A female attached to the army, newly arrived from Africa, and enjoying perfect health, undertook to nurse an infant residing at Toulon; on the third day of the nursing, a marsh fever attacked the infant, and did not yield but to the employment of quinine."

Treatment. The therapeutic portion of the work is devoted chiefly to the virtues of arsenic. As a remedial agent, this substance has been employed from remote antiquity, according to Homberg, even by the Chinese and Hindoos. It has been long employed in modern times by all the nations of Europe. Our author, after some comments on the *doses* in which this remedy has been administered by different authors, viz. $\frac{1}{8}$ or $\frac{1}{2}$ grain, remarks as follows:

"It is because I am less convinced than these authors of the entire innocency of arsenical preparations administered in such doses, that I have applied myself to discover if it be not possible to preserve their efficacy by a smaller dose, while at the same time depriving them of their poisonous inconveniences; and I believe I have succeeded in resolving this problem in the most satisfactory manner. After having commenced my experiments with the twenty-fourth part of a grain, I am assured by successive trials, which have been already repeated with similar results by a good number of physicians at Marseilles, that arsenious acid properly prepared preserves, in the somewhat microscopic dose of the hundredth of a grain (un demi-milligramme), all its medicinal energy, not only in the treatment of marsh fevers, but also in a multitude of other diseases. Further; I have often obtained by a single dose of the hundredth of a grain of this medicine, the entire removal of fevers contracted in Algeria and Senegal, and which had previously resisted means of various kinds, including the sulphate of quinine and change of climate. .......... A very remarkable circumstance is that the degree of efficacy of arsenical preparations, in the treatment of intermittent fevers, is subordinate manifestly to the reigning medical constitution, so that they are seen sometimes to lose in a great degree their febrifuge virtue, although some days previously, no intermittent fever resisted their heroic action." (pp. 276-8.)

This circumstance he ascertained not to depend on errors in preparing the material. The sulphate of quinine sometimes produced the best effects when the condition specified above occurred; but in the great majority of cases, the quinine also failed when the arsenious acid did not succeed. He has ascertained by numerous experiments that the action of quinine is equally influenced by the epidemic constitution. On the whole, in respect to the comparative febrifuge virtues of quinine and arsenic, he has found that the success of the former when the latter had failed formed the exception to the general rule, while nothing was more common than to find the arsenic successful in cases which resisted the quinine.

"I have been able, in a great number of cases, and by very small doses of arsenious acid, to put an end in a short time to quotidian, tertian, and quartan fevers, contracted in latitudes the most various, often complicated with chronic engorgement of the abdominal viscera, and for a long time rebellious to the sulphate of quinine." (p. 280.)

A review of the treatment of 266 cases, only half of those in which he had administered arsenical preparations, of which he has preserved notes, gives the following results: Of fevers, quotidian, tertian, quartan, quintan, irregular, and masked, 188 which had not undergone previous treatment, were cured by arsenic; 57 which resisted quinine were cured by arsenic; 13 which resisted arsenic were cured by quinine; and 8 resisted both remedies. He cannot specify the circumstances which would *à priori* indicate the preference of arsenic to quinine; but he has always begun his treatment with the former, and has had recourse to the latter when the two or three first doses of the arsenic had not produced the desired result. He has used arsenic with success on many occasions in continued forms of disease when these were capable of being traced to marsh poisoning. Its beneficial action is not confined, however, to diseases which originated from this cause, for he has found it to have the power, in the dose of one hundredth of a grain of arsenious acid, of removing the paroxysmal complication which is so often noticed in the typhoid fever; in which disease it also appears to exert a contra-stimulant effect.

In considering the mode in which arsenic acts as a medicinal agent, he justly distinguishes its irritant effects on the gastro-intestinal mucous membrane from its remedial properties. The latter he ascribes to its absorption into the blood, an event which is favoured by the absence of its irritant effects, which latter interfere with the due performance of absorption,—whence his preference of minute doses.

M. Boudin is evidently puzzled between his doctrines of antagonism and a certain leaning to homœopathy. He mentions at p. 264, that on one occasion the arsenic appeared to produce, in a patient to whom he administered it for the cure of ichthyosis, a quotidian intermittent fever, which he was obliged to subdue with quinine; and at p. 296 he says: "On meditating on the phenomena observed in a great number of individuals poisoned by arsenic, and of which authors have transmitted to us an account, it is difficult not to perceive a certain analogy between these phenomena and those which the medicine is often required to combat in the sick."

In treating intermittent fevers with arsenic, he thinks it of great consequence that the doses should be administered always five or six hours before an expected paroxysm. He abstains from giving the medicine on the days of apyrexia, as useless; and if after two or three successive administrations no effect is produced, he resorts to quinine. In the continued form of marsh fevers he does not specify any particular time for the doses, nor does he mention the frequency with which they should be repeated. In cases complicated with bilious or inflammatory disorders, he advises the discontinuance of the remedy, or its being assisted by other means. In old and obstinate intermittents it may be continued for a longer time than is already specified; but no precise directions are given on this head.

The preparation which he prefers is the arsenious acid, from the greater simplicity of its composition than that of the other compounds of the metal. This or any other preparation may be given in the form of powder, with the sugar of milk; in pills; lavements; cigars (*cigarettes*), so as to be absorbed by the bronchial mucous membrane; or ointments. The cigarettes are much of the same kind as those employed by Trousseau in pulmonary disease. Boudin recommends an hundredth part of a grain of arsenious acid to be deposited on a small piece of paper, and then moistened with a few drops of water, so that the paper may absorb the solution. The paper being dried, rolled up, and lighted, the inhalation is performed. He does not say whether he has found this plan succeed in marsh fevers, the note to his formula containing directions merely for cases of asthma. If the arsenic be required for infants, he says, it may be given to the nurse or to a she-goat, or she-ass, whereby the milk becomes furnished with

LIBRARY OF THE

Orleans Parish Medical Society the properties of the medicine. It would be difficult to regulate the quantity in this way, we suspect.

Of auxiliary remedies M. Boudin says very little. Arsenic needs them much less frequently than quinine does. He refers to the occasional necessity of emetics in bilious complications, and of bloodletting in the inflammatory.

The high price and frequent adulteration of quinine render such a remedy as arsenic worthy of much more attention than has been hitherto bestowed on it. In military practice more especially we are surprised that it has not come into more general use, considering that its influence in marsh diseases has been so long established. One great objection has, doubtless, been the occasional violence of what Fowler calls its "operative effects," when administered in the doses which have been commonly prescribed; and therefore, presuming that M. Boudin's account of the advantage to be derived from minute doses is correct, we consider his suggestions on this point as extremely important, and receive them thankfully, as some atonement for the poverty of his book in other respects.

---

